# Supplementary material for: Genome-Wide Identification and Expression Analysis of the Aux/IAA Gene Family of the Drumstick Tree (Moringa oleifera Lam.) Reveals Regulatory Effects on Shoot Regeneration
Source: Int J Mol Sci. 2022 Dec 11;23(24):15729. doi: 10.3390/ijms232415729 (PMC9779525; doi:10.3390/ijms232415729)
Supplement: Supplementary file 1 [file ijms-23-15729-s001.zip › Supplemental Figure S1.pdf]

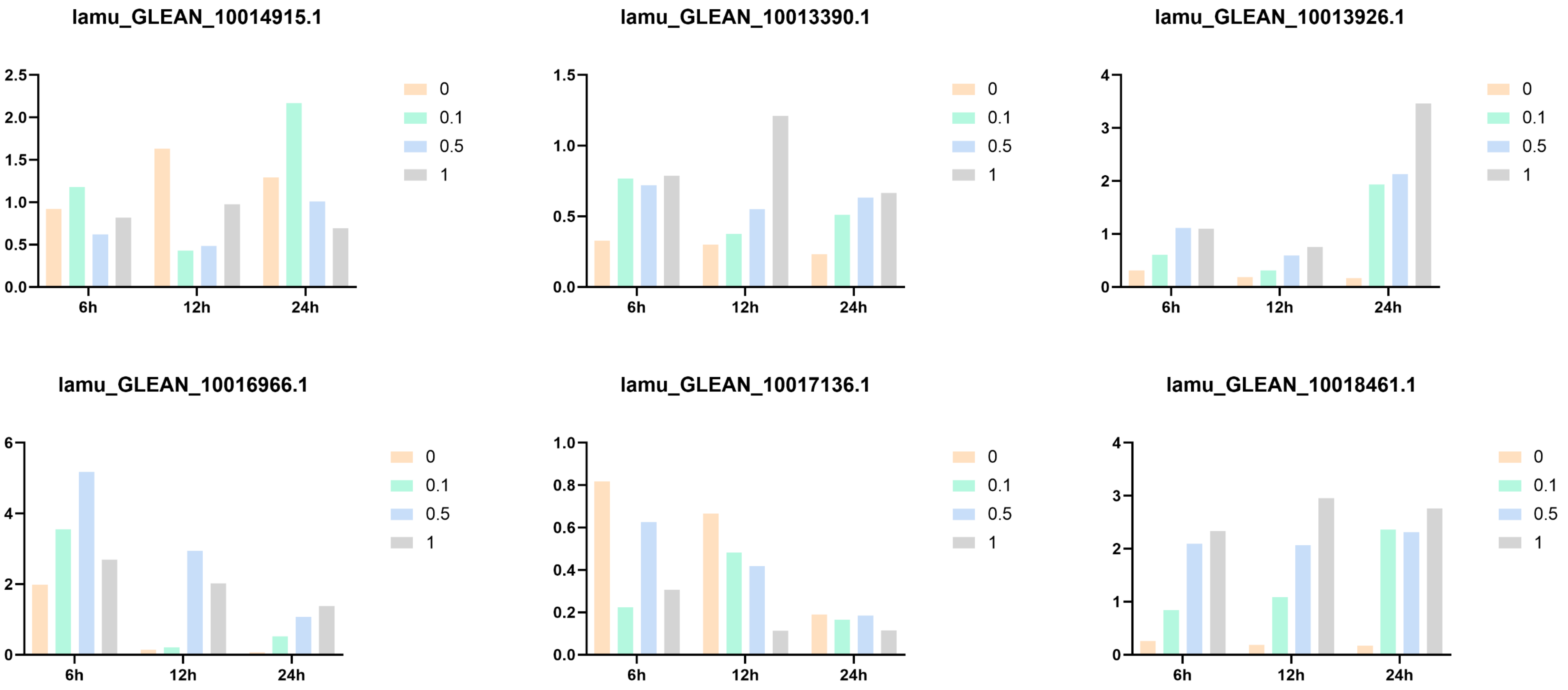
lamu_GLEAN_10014915.1
lamu_GLEAN_10013390.1
lamu_GLEAN_10013926.1
lamu_GLEAN_10016966.1
lamu_GLEAN_10017136.1
lamu_GLEAN_10018461.1
6h
12h
24h
0
0.1
0.5
1

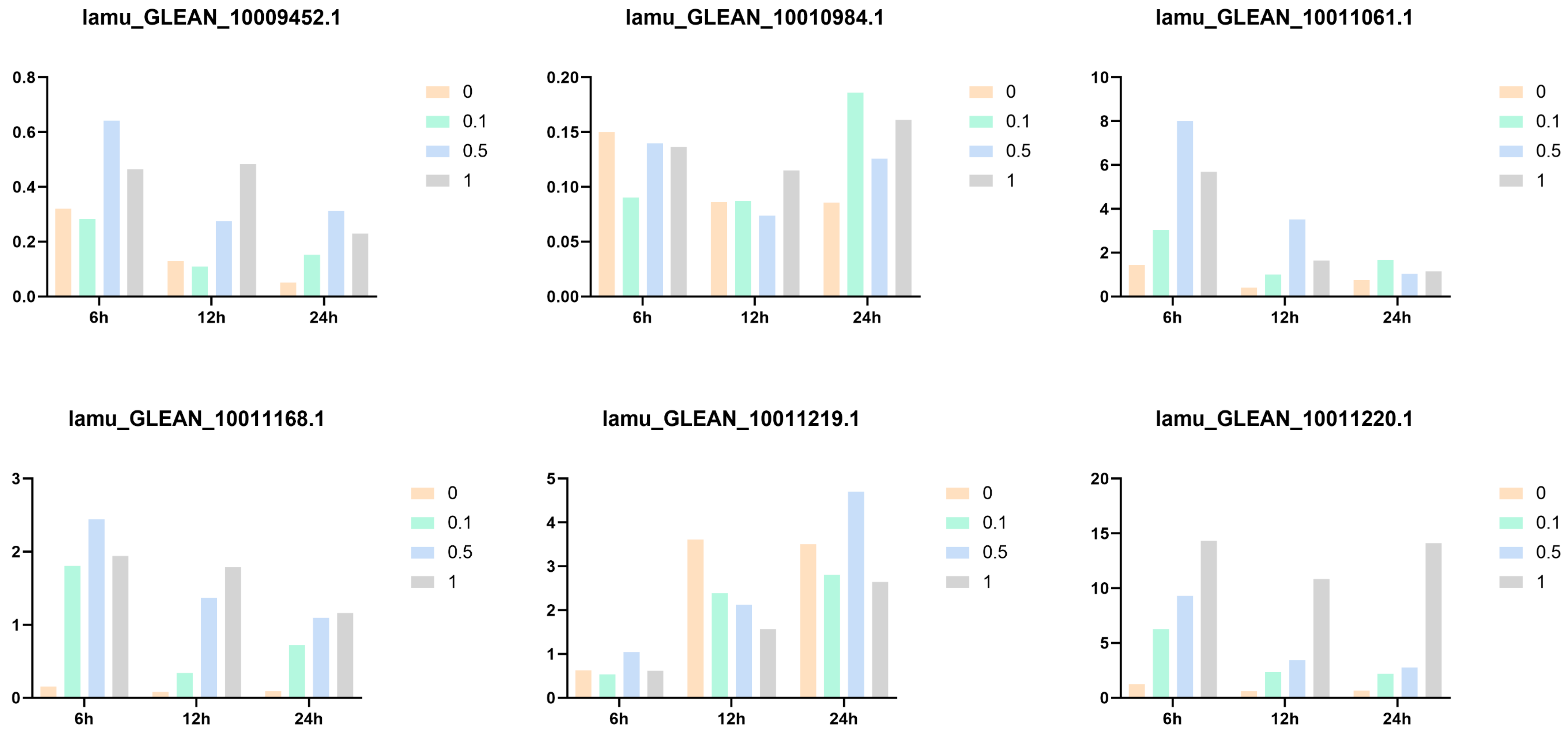
lamu_GLEAN_10009452.1
0.8
0.6
0.4
0.2
0.0
6h
12h
24h
0
0.1
0.5
1
lamu_GLEAN_10010984.1
0.20
0.15
0.10
0.05
0.00
6h
12h
24h
0
0.1
0.5
1
lamu_GLEAN_10011061.1
10
8
6
4
2
0
6h
12h
24h
0
0.1
0.5
1
lamu_GLEAN_10011168.1
3
2
1
0
6h
12h
24h
0
0.1
0.5
1
lamu_GLEAN_10011219.1
5
4
3
2
1
0
6h
12h
24h
0
0.1
0.5
1
lamu_GLEAN_10011220.1
20
15
10
5
0
6h
12h
24h
0
0.1
0.5
1

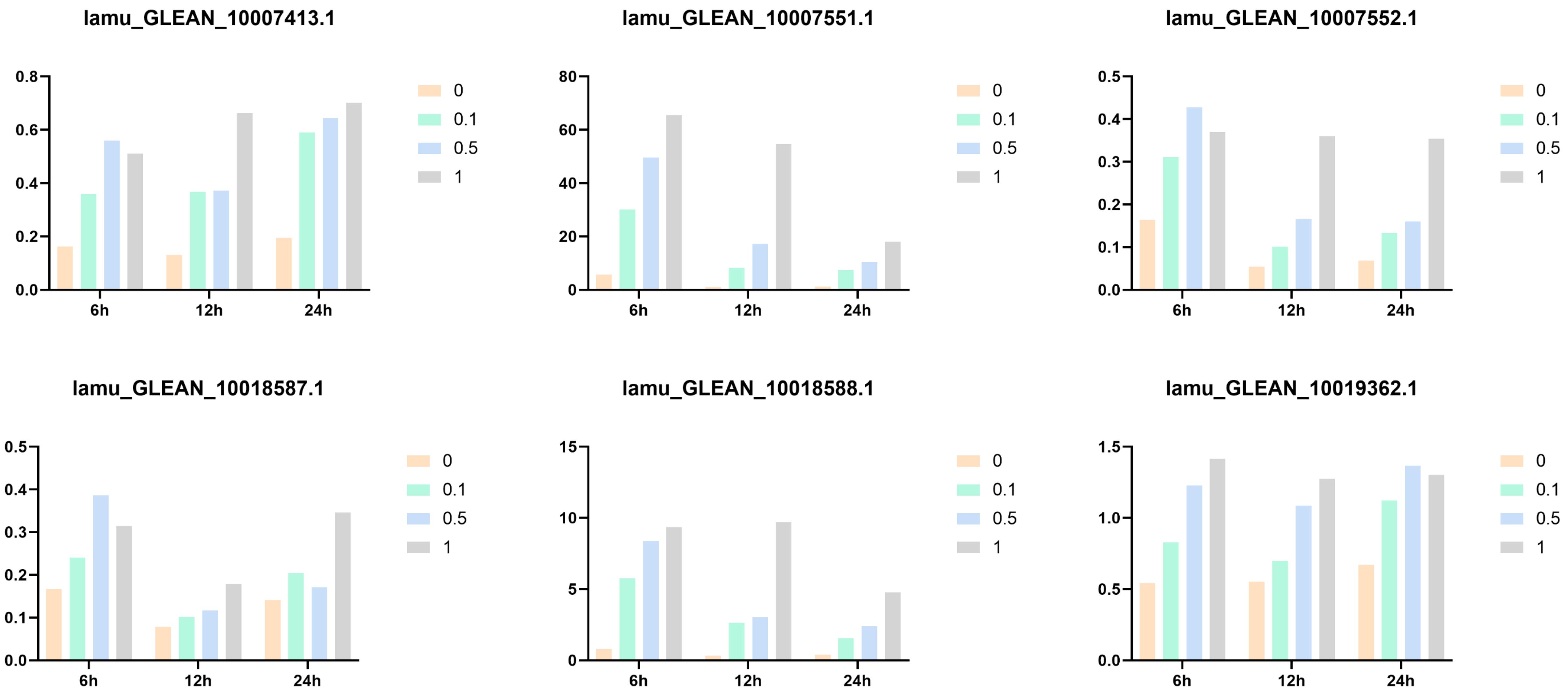
lamu_GLEAN_10007413.1
0.0
0.2
0.4
0.6
0.8
6h
12h
24h
0
0.1
0.5
1
lamu_GLEAN_10007551.1
0
20
40
60
80
6h
12h
24h
0
0.1
0.5
1
lamu_GLEAN_10007552.1
0.0
0.1
0.2
0.3
0.4
0.5
6h
12h
24h
0
0.1
0.5
1
lamu_GLEAN_10018587.1
0.0
0.1
0.2
0.3
0.4
0.5
6h
12h
24h
0
0.1
0.5
1
lamu_GLEAN_10018588.1
0
5
10
15
6h
12h
24h
0
0.1
0.5
1
lamu_GLEAN_10019362.1
0.0
0.5
1.0
1.5
6h
12h
24h
0
0.1
0.5
1

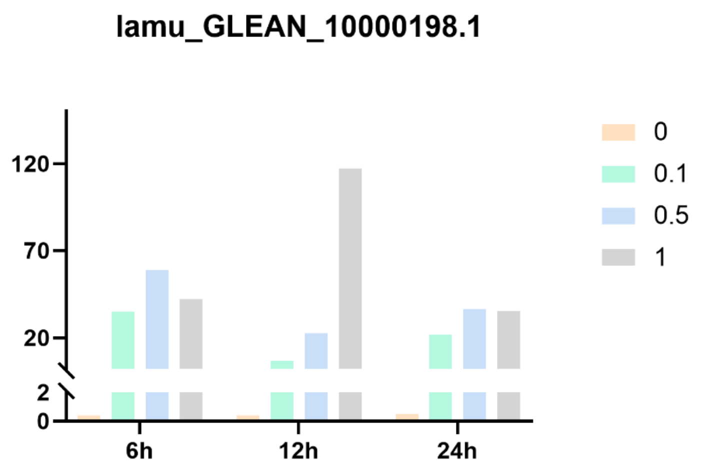
lamu_GLEAN_10000198.1
120
70
20
2
0
6h
12h
24h
0
0.1
0.5
1

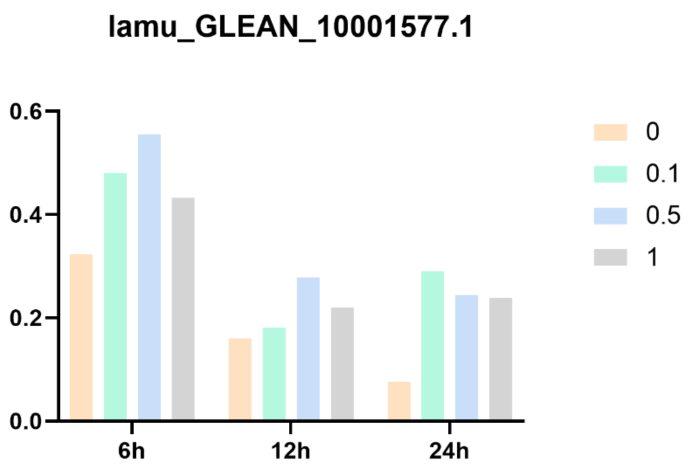
lamu_GLEAN_10001577.1
0.6
0.4
0.2
0.0
6h
12h
24h
0
0.1
0.5
1

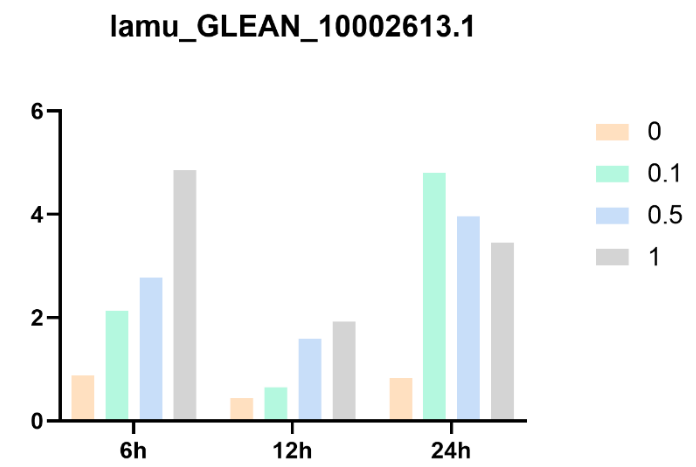
lamu_GLEAN_10002613.1
6
4
2
0
6h
12h
24h
0
0.1
0.5
1

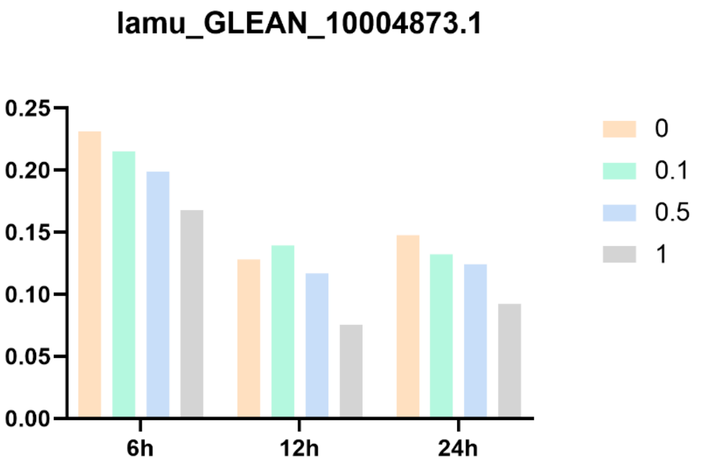
lamu_GLEAN_10004873.1
0.25
0.20
0.15
0.10
0.05
0.00
6h
12h
24h
0
0.1
0.5
1

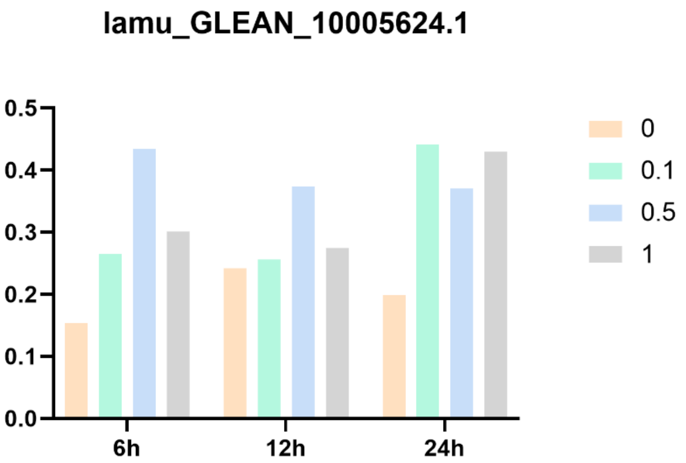
lamu_GLEAN_10005624.1
0.5
0.4
0.3
0.2
0.1
0.0
6h
12h
24h
0
0.1
0.5
1
