# Supplementary material for: Genome-Wide Identification and Expression Analysis of the Aux/IAA Gene Family of the Drumstick Tree (Moringa oleifera Lam.) Reveals Regulatory Effects on Shoot Regeneration
Source: Int J Mol Sci. 2022 Dec 11;23(24):15729. doi: 10.3390/ijms232415729 (PMC9779525; doi:10.3390/ijms232415729)
Supplement: Supplementary file 1 [file ijms-23-15729-s001.zip › Supplemental Table S1.pdf]

Gene ID	Forward Primer	Reverse Primer
ACP2	GAAACCAATGAGCACCCAGC	GATGAATACCAGTCCACCGCAAC
lamu_GLEAN_10000198.1	GAAGAACAGTTTCAGCGACAA	TGACTCACCGATGCCATAA
lamu_GLEAN_10001577.1	GCAGTTCAAGAGACGAAAAAT	CTTGCGGAAGGAACGGATT
lamu_GLEAN_10002613.1	CAAAGGCTCAAGTAGTGGGG	GGGGTCCTCGGTGCTAATCC
lamu_GLEAN_10004873.1	ATTCAAGCCTCGCACAACA	CACGCATTCAACGAACTCC
lamu_GLEAN_10005624.1	ACAACCCTGTTGCACTAAA	TTGCCAGATTCTTCCTAAA
lamu_GLEAN_10007413.1	GGATGGTGCTCCTTATTTG	CGTATTGACCTATGGTAAAAC
lamu_GLEAN_10007551.1	TTGCCAGGAAGCGATGAGC	CATCCCACCACTTGTGCCTT
lamu_GLEAN_10007552.1	AAGCCCTTGCTCAACGACA	CGGAATGACCGAACTGGTG
lamu_GLEAN_10009452.1	TGGTCTAACTGTCCTGGTG	AAGAGTGATTTGATGGGTG
lamu_GLEAN_10010984.1	GGACGATGCTGATAAGAACG	CCACCGCCACCACTACAAC
lamu_GLEAN_10011061.1	CACTGGTAGGAGGAACTGC	CATCTACGGCGACCTTTAC
lamu_GLEAN_10011168.1	TCCAACTGGGTTCGTCTTT	GGTTCCTCGGGTCATCTTT
lamu_GLEAN_10011219.1	GCTCTACGTGGGACGCTA	CACCTTCAGGCATTTACCG
lamu_GLEAN_10011220.1	CACGGAGTTGAGATTAGGG	TCTTCATCGGATTTCTTGG
lamu_GLEAN_10013390.1	CTACAGAACTGAGGCTTGG	AAGGAGTGGAAATAGTGGC
lamu_GLEAN_10013926.1	CCCTTTGATACCAAGTGAA	AGCCAGCAGGTCTAGTTTT
lamu_GLEAN_10014915.1	CCCTGGCTTTGACGATGAC	GCTTTAGCGAGGCTTTGGT
lamu_GLEAN_10016966.1	CAACCACCCGCAGTTCTCC	AAAGCAAACCCACCATTCG
lamu_GLEAN_10017136.1	CTTCTGGCTCTTCCCTTAT	CTGCTGCTTGCTAGATTTT
lamu_GLEAN_10018461.1	TGGAGGCTACTGAGGCTAG	TGACAGACCACAGAAGGGA
lamu_GLEAN_10018587.1	ATCTCCTCCCTGCTGCTTG	CTTGACTTGTCGCCCTCTT
lamu_GLEAN_10018588.1	GTGGCGTATGAGAATGACC	GCTTGTTTCCAGCAAAGAC
lamu_GLEAN_10019362.1	TTGGATTGAGTCTTGGTGGTG	GGTCCCAGCGGTGACATTA
