# Supplementary material for: Genome-Wide Identification and Expression Analysis of the Aux/IAA Gene Family of the Drumstick Tree (Moringa oleifera Lam.) Reveals Regulatory Effects on Shoot Regeneration
Source: Int J Mol Sci. 2022 Dec 11;23(24):15729. doi: 10.3390/ijms232415729 (PMC9779525; doi:10.3390/ijms232415729)
Supplement: Supplementary file 1 [file ijms-23-15729-s001.zip › Supplemental Table S2.pdf]

Primer name	Sequence
lamu_GLEAN_10011219-F	ATGGGTTCGGAGCGAGAA
lamu_GLEAN_10011219-R	ACTTCTGTTCTTGCATTT
lamu_GLEAN_10007551-F	ATGGGTAAGTCTTTGGCTTT
lamu_GLEAN_10007551-R	TAGAGAAGTCAAGCCTCTA
lamu_GLEAN_10000198-F	ATGGCGATCGCAGCTG
lamu_GLEAN_10000198-R	GAAAGAAGGTGGTGACTC
