# Supplementary material for: Genome-Wide Identification and Expression Analysis of the Aux/IAA Gene Family of the Drumstick Tree (Moringa oleifera Lam.) Reveals Regulatory Effects on Shoot Regeneration
Source: Int J Mol Sci. 2022 Dec 11;23(24):15729. doi: 10.3390/ijms232415729 (PMC9779525; doi:10.3390/ijms232415729)
Supplement: Supplementary file 1 [file ijms-23-15729-s001.zip › Supplemental Table S3.pdf]

>AtIAA10
MNGLQEVCSSSGSVMIGLPAEEDENAAHSSEDSSCPDESVSETELDLALGLSIGRRKVRSSLSSSSSSLTRESGTK
RSADSSPAAASNATRQVAVGWPPL
RTYRINSLVNQAKSLATEGGLSSGIQKETTKSVVVAAKNDDACFIKSSRTSMLVKVTMDGVIIGRKVDLNALDSY
AALEKTLDLMFFQIPSPVTRSNTQG
YKTIKETCTSKLLDGSSEYIITYQDKDGDWMLVGDVPWQMFLGSVTRLRIMKTSIGAGVGK*
>AtIAA3
MDEFVNLKETELRLGLPGTDNVCEAKERVSCCNNNNKRVLSTDTEKEIESSSRKTETSPPRKAQIVGWPPVRSY
RKNNIQSKKNESEHEGQGIYVKVSMD
GAPYLRKIDLSCYKGYSELLKALEVMFKFSVGEYFERDGYKGSDFVPTYEDKDGDWMLIGDVPWEMFICTCKRL
RIMKGSEAKGLGCGV*
>AtIAA17
MMGSVELNLRETELCLGLPGGDTVAPVTGNKRGFSETVDLKLNLNNEPANKEGSTTHDVVTFDSKEKSACPKD
PAKPPAKAQVVGWPPVRSYRKNVMVSC
QKSSGGPEAAAFVKVSMDGAPYLRKIDLRMYKSYDELSNALSNMFSSFTMGKHGGEEGMIDFMNERKLMDL
VNSWDYVPSYEDKDGDWMLVGDVPWPMFV
DTCKRLRLMKGSDAIGLAPRAMEKCKSRA*
>AtIAA12
MRGVSELEVGKSNLPAESELELGLGLSLGGGAWKERGRILTAKDFPSVGSKRSAESSSHQGASPPRSSQVVGWP
PIGLHRMNSLVNNQAMKAARAEEGDG
EKKVVKNDELKDVSMKVNPKVQGLGFVKVNMDGVGIGRKVDMRAHSSYENLAQTLEEMFFGMTGTTCREK
VKPLRLLDGSSDFVLTYEDKEGDWMLVGDV
PWRMFINSVKRLRIMGTSEASGLAPRRQEQKDRQRNNPV*
>AtIAA34
MYCSDPPHPLHLVASDKQQKDHKLILSWKKPTMDSDPLGVFPNSPKYHPYYSQTTEFGGVIDLGLSLRTIQHEIY
HSSGQRYCSNEGYRRKWGYVKVTMD
GLVVGRKVCVLDHGSYSTLAHQLEDMFGMQSVSGLRLFQMESEFCLVYRDEEGLWRNAGDVPWNEFIESVER
LRITRRNDAVLPF*
>AtIAA5
MANESNNLGLEITELRLGLPGDIVVSGESISGKKRASPEVEIDLKCEPAKKSQVVGWPPVCSYRRKNSLERTKSSY
VKVSVDGAAFLRKIDLEMYKCYQD
LASALQILFGCYINFDDTLKESECVPIYEDKDGDWMLAGDVPWEMFLGSCKRLRIMKRSCNRG*
>AtIAA18
MEGYSRNGEISPKLLDLMIPQERRNWFHDEKNSVFKTEEKKLELKLGPPGEEDDDESMIRHMKKEPKDKSILSL
AGKYFSPSSTKTTSHKRTAPGPVVGW
PPVRSFRKNLASGSSSKLGNDSTTSNGVTLKNQKCDAAAKTTEPKRQGGMFVKINMYGVPIGRKVDLSAHNSY
EQLSFTVDKLFRGLLAAQRDFPSSIED
EKPITGLLDGNGEYTLTYEDNEGDKMLVGDVPWQMFVSSVKRLRVIKTSEISSALTYGNGKQEKMRR*
>AtIAA6
MAKEGLALEITELRLGLPGDNYSEISVCGSSKKKKRVLSDMMTSSALDTENENSVVSSVEDESLPVVKSQAVGW
PPVCSYRRKKNNEEASKAIGYVKVSM
DGVPYMRKIDLGSSNSYINLVTVLENLFGCLGIGVAKEGKKCEYIIIYEDKDRDWMLVGDVPWQMFKESCKRLRI
VKRSDATGFGLQQD*
>AtIAA15

MSPEEYVRVWPDSGDLGGTELTLALPGTPTNASEGPKKFGNKRRFLETVDLKLGEAHENNYISSMVTNDQLVG
WPPVATARKTVRRKYVKVALDGAAYLR
KVDLGMYDCYGQLFTALENMFQGIITICRVTELERKGEFVATYEDKDGDLMLVGDVPWMMFVESCKRMRLM
KTGDAIGL*
>AtIAA32
MDPNTPADFFKGSSKFHTYYSQTKKGGGVIDLGLSLRTIQHETYLPPARMIGLDGYGELIDWSQPSYNSITQLKS
EDTGHQRLAQGYYNNEGESRGKYAY
VKVNLDGLVVGRKVCLVDQGAYATLALQLNDMFGMQTVSGLRLFQTESEFSLVYRDREGIWRNVGDVPWKEF
VESVDRMRIARRNDALLPF*
>AtIAA8
MSSGNDKIKQVLHIEDLMSYRLLSVDKDELVTSPCLKERNYLGLSDCSSVDSSTIPNVVGKSNLNFKATELRLGLP
ESQSPERETDFGLLSPRTPDEKLL
FPLLPSKDNGSATTGHKNVVSGNKRGFADTWDEFSGVKGSVRPGGGINMMLSPKVKDVSKSIQEERSHAKGG
LNNAPAAKAQVVGWPPIRSYRKNTMASS
TSKNTDEVDGKPGLGVLFVKVSMDGAPYLRKVDLRTYTSYQQLSSALEKMFSCFTLGQCGLHGAQGRERMSEI
KLKDLLHGSEFVLTYEDKDGDWMLVGD
VPWEIFTETCQKLKIMKGSDSIGLAPGAVEKSKNKERV*
>AtIAA13
MITELEMGKGESELELGLGLSLGGGTAAKIGKSGGGGAWGERGRLLTAKDFPSVGSKRAADSASHAGSSPPRSS
SQVVGWPPIGSHRMNSLVNNQATKSA
REEEEAGKKKVKDDEPKDVTKKVNGKVQVGFIKVNMDGVAIGRKVDLNAHSSYENLAQTLEDMFFRTNPGTV
GLTSQFTKPLRLLDGSSEFVLTYEDKEG
DWMLVGDVPWRMFINSVKRLRVMKTSEANGLAARNQEPNERQRKQPV*
>AtIAA20
MGRGRSSSSSSIESSSKSNPFGASSSTRNLSTDLRLGLSFGTSSGTQYFNGGYGYSVAAPAVEDAEYVAAVEEEEE
NECNSVGSFYVKVNMEGVPIGRKI
DLMSLNGYRDLIRTLDFMFNASILWAEEEDMCNEKSHVLTYADKEGDWMMVGDVPWEMFLSTVRRLKISRA
NYHY*
>AtIAA16
MINFEATELRLGLPGGNHGGEMAGKNNGKRGFSETVDLKLNLSSTAMDSVSKVDLENMKEKVVKPPAKAQV
VGWPPVRSFRKNVMSGQKPTTGDATEGND
KTSGSSGATSSASACATVAYVKVSMDGAPYLRKIDLKLYKTYQDLSNALSKMFSSFTIGNYGPQGMKDFMNESK
LIDLLNGSDYVPTYEDKDGDWMLVGD
VPWEMFVDSCKRIRIMKGSEAIGLAPRALEKCKNRS*
>AtIAA19
MEKEGLGLEITELRLGLPGRDVAEKMMKKRAFTEMNMTSSGSNSDQCESGVVSSGGDAEKVNDSPAAKSQV
VGWPPVCSYRKKNSCKEASTTKVGLGYVK
VSMDGVPYLRKMDLGSSQGYDDLAFALDKLFGFRGIGVALKDGDNCEYVTIYEDKDGDWMLAGDVPWGMF
LESCKRLRIMKRSDATGFGLQPRGVDE*
>AtIAA26
MEGCPRNREIGPKLLDLIPQGRKWYQEDKNNTDQEKKLELRLGPPGGDEEDHSAIKKKNTEIRNIKKETEDKSF
HCFNGNHFSPSNKTTSVPHISQKRTA
PGPVVGWPPVRSFRKNLASTSSSKLGNESSHGGQINKSDDGEKQVETKKEGMFVKINMDGVPIGRKVDLNAY
NSYEQLSFVVDKLFRGLLAAQRDISDGQ

GEEKPIIGLLDGKGEFTLTYEDNEGDKMLVGDVPWQMFVSSVKRLRVIKSSEISSALTFGCSKQEKMMH*
>AtIAA31
MEVSNSCSSFSSSSVDSTKPSPSESSVNLSLSLTFPSTSPQREARQDWPPIKSRLRDTLKGRRLLRRGDDTSLFVK
VYMEGVPIGRKLDLCVFSGYESLL
ENLSHMFDTSIICGNRDRKHHVLTYEDKDGDWMMVGDIPWDMFLETVRRLKITRPERY*
>AtIAA2
MAYEKVNELNLKDTELCLGLPGRTEKIKEEQEVSCVKSNNKRLFEETRDEEESTPPTKTQIVGWPPVRSSRKNNN
SVSYVKVSMDGAPYLRKIDLKTYKN
YPELLKALENMFKVMIGEYCEREGYKGSGFVPTYEDKDGDWMLVGDVPWDMFSSSCKRLRIMKGSDAPALDS
SL*
>AtIAA7
MIGQLMNLKATELCLGLPGGAEAVESPAKSAVGSKRGFSETVDLMLNLQSNKEGSVDLKNVSAVPKEKTTLKDP
SKPPAKAQVVGWPPVRNYRKNMMTQQ
KTSSGAEEASSEKAGNFGGGAAGAGLVKVSMDGAPYLRKVDLKMYKSYQDLSDALAKMFSSFTMGNYGAQG
MIDFMNESKLMNLLNSSEYVPSYEDKDGD
WMLVGDVPWEMFVESCKRLRIMKGSEAVGLAPRAMEKYCKNRS*
>AtIAA30
MGRGRSSSSSSIESSCKSNPFGVSSSNTRNLSTDLRLGLSFGSSSGQYYNGGDNHEYDGVGAAEEMMIMEEEE
QNECNSVGSFYVKVNMEGVPIGRKIDL
LSLNGYHDLITTLDYMFNASILWAEEEDMCSEKSHVLTYADKEGDWMMVGDVPWEMFLSSVRRLKISRAYHY
*
>AtIAA14
MNLKETELCLGLPGGTETVESPAKSGVGNKRGFSETVDLKLNLQSNKQGHVDLNTNGAPKEKTFLKDPSKPPAK
AQVVGWPPVRNYRKNVMANQKSGEAE
EAMSSGGGTVAFVKVSMDGAPYLRKVDLKMYTSYKDLSDALAKMFSSFTMGSYGAQGMIDFMNESKVMDL
LNSSEYVPSYEDKDGDWMLVGDVPWPMFVE
SCKRLRIMKGSEAIGLAPRAMEKFKNRS*
>AtIAA1
MEVTNGLNLKDTELRLGLPGAQEEQQLELSCVRSNNKRKNNDSTEESAPPPAKTQIVGWPPVRSNRKNNNNK
NVSYVKVSMDGAPYLRKIDLKMYKNYPE
LLKALENMFKFTVGEYSEREGYKGSGFVPTYEDKDGDWMLVGDVPWDMFSSSCQKLRIMKGSEAPTAL*
>AtIAA11
MEGGSASGSASALSNDENLVVSCEDSSSPIGNELELGLTLSLGRKGYRDCRVYADDSSSSSSSSLSRASVIAGIKR
TADSMAATSGQVVGWPPIRTYRM
NSMVNQAKASATEDPNLEISQAVNKNRSDSTKMRNSMFVKVTMDGIPIGRKIDLNAHKCYESLSNTLEEMFLK
PKLGSRTLETDGHMETPVKILPDGSSG
LVLTYEDKEGDWMLVGDVPWGMFIGSVRRLRIMKTSEATGKDDIMKQIIIYEEPFMFEAVIRQITDQREDKNIV
RSFFFFSPLYSFFFGSAIFLLVSYMF
SL*
>AtIAA27
MSVSVAAEHDYIGLSEFPTMEATTMSDKTKTRDNNNGLNFKATELRLGLPGSESPERVDSRFLALNKSSCPVSG
AKRVFSDAINDSNKWVFSPGSTTATG
DVGSGSGPRTSVVKDGKSTTFTKPAVPVKEKKSSATAPASKAQVVGWPPIRSFRKNSMASSQSQKPGNNSETE
EAEAKSGPEQPCLYVKVSMEGAPYLRK

IDLKTYKSYLELSSALEKMFSCFTIGQFGSHGGCGRDGLNESRLTDLLRGSEYVVTYEDKDSDWMLVGDVPWE
MFICSCKKLRIMKSSEAIGLAPRVMEK
CRSRN*
>AtIAA29
MELDLGLSLSPHKSSKLGFNFDLNKHCAIEGAASCLGTEKLRFEATFGLGNVEENCYMPKQRLFALNGQPNEED
EDPLESESSIVYDDEEENSEVVGWPP
VKTCMIKYGSYHHRHIRNHHHCPYHHRGRRITAMNNNISNPTTATVGSSSSSSISSRSSMYVKVKMDGVAIARK
VDIKLFNSYESLTNSLITMFTEYEDC
DREDTNYTFTFQGKEGDWLLRGDVTWKIFAESVHRISIIRDRPCAYTRCLF*
>AtIAA28
MEEEKRLELRLAPPCHQFTSNNNINGSKQKSSTKETSFLSNNRVEVAPVVGWPPVRSSRRNLTAQLKEEMKKKE
SDEEKELYVKINMEGVPIGRKVNLSA
YNNYQQLSHAVDQLFSKKDSWDLNRQYTLVYEDTEGDKVLVGDVPWEMFVSTVKRLHVLKTSHAFSLSPRKH
GKE*
>AtIAA4
MEKVDVYDELVNLKATELRLGLPGTEETVSCGKSNKRVLPEATEKEIESTGKTETASPPKAQIVGWPPVRSYRKN
NVQTKKSESEGQGNYVKVSMDGAPY
LRKIDLTMYKQYPELMKSLENMFKFSVGEYFEREGYKGSDFVPTYEDKDGDWMLVGDVPWEMFVSSCKRLRI
MKGSEVKGLGCGGL*
>AtIAA33
MNSFEPQSQDSLQRRFHQDNSTTQQPRDTTTPFIPKPASKNHNNSNSSSGAAGRSFQGFGLNVEDDLVSSVV
PPVTVVLEGRSICQRISLDKHGSYQSLA
SALRQMFVDGADSTDDLDLSNAIPGHLIAYEDMENDLLLAGDLTWKDFVRVAKRIRILPVKGNTRQVKRNE*
>AtIAA9
MSPEEELQSNVSVASSSPTSNCISRNTLGGLKEHNYLGLSDCSSVGSSTLSPLAEDDKATISLKATELTLGLPGSQS
PARDTELNLLSPAKLDEKPFFPL
LPSKDEICSSSQKNNASGNKRGFSDTMDQFAEAKSSVYTEKNWMFPEAAATQSVTKKDVPQNIPKGQSSTTN
NSSSPPAAKAQIVGWPPVRSYRKNTLAT
TCKNSDEVDGRPGSGALFVKVSMDGAPYLRKVDLRSYTNYGELSSALEKMFTTFTLGQCGSNGAAGKDMLSE
TKLKDLLNGKDYVLTYEDKDGDWMLVGD
VPWEMFIDVCKKLKIMKGCDAIGLAAAPRAMEKSKMRA*
>lamu_GLEAN_10000198
MAIAAAGVEITTELRLGLPGDDHQGFKDKIVNEKKRVFSEMSDDGSVVTSDDNRQVSAKGQVVGWPPVCTY
RRKNSFSDKDCTETSKMYVKVSMDGAPFLRKIDLGMQKGYSDLAMALEKLFGCYGIGESPPSF*
>lamu_GLEAN_10001577
MSTQLEHDYIGLRDTSPMERSSDKISSSSSSSSFPTLPNEDNNKNSKNSGSIGLNLKETELRLGLPGSQSPERKSA
HGVSLFGKDLDQNTNGYSLGPLKNLVSGAKRGFSDAIDGSSGKWVFSKSEVDMSKGAVLFSPRGVVENKSNTH
HSCVAKEIGVVPQSPKAVQETKNQESVANEHAGAPASKAQVVGWPPIRSFRKNTMASNLGKNNDDSEGKSG
SGCIYVKVSMDGAPYLRKVDLNTYDNYSQLSSALEKMFSCFTIGQCGSKDGLSESRLVDLLHGSEFVLTYEDKDG
DWMLVGDVPWEMFTDTCRRLRIMKGSEAIGLAPRAMEKCKGSES*
>lamu_GLEAN_10002613
MSLENGTCLPESGNAVLNFKATELTLGLPGEHRATSDGTAKSGVKRGFLETVDLNLESSELNYDYRNCKKSENHA
ASSVHKLPAPKAQVVGWPPVRGYRKNAMKSCKYVKVAVDGAPYLRKIVRGVLQAAAADEKQRGNWISTEDP
SKMLKHKLKSSP*

>lamu_GLEAN_10004873
MDSNASGFLWNTSNLHHPVYYQGKEDDGIIDLGLSLRTAHPEADPPSGHMPSLEGYSELIGWPQANSQLKNSI
TGYKKLLQEDCSDEAGVQSKDRWAYVKVNMDGVMVGRKVCLLAHGGYSSLAQQLEDMFGRKSASELRLFHP
GSEFALIYKDKEENWRTAGDIPWKEFVECVKRLRIARRSDVLSSCSSPFK*
>lamu_GLEAN_10005624
MEGCSKSGEACPQLLDLITKERQWLVNRDEKRSHGSSEEKKLELRLGPPGEDWSIQGTAKSNFIERDDRSLLSLG
YFSSMNNNTNNGNQTHEFSLPENHPVGSVLSAPWTQQHNQQKTTTSTKAPSFLQFSSTGTTVPQGLPVMVK
ESSQPCCTKVVDLQNAEKKAFSPSPANTAVPNSSQKRTAPGPVVGWPPIRSFRKNLASSSSSKPATESPNVVQN
NVASEKPVEPNRKGLFVKINMDGVPIGRKVDLKSYDSYEKLSTAVDELFRGLLAAQGDLPAGGIMNKPEGEKSIT
GVLDGSGEYTLVYEDNEGDRMLVGDVPWHMFVSTVKRLRVLKSSELSVLSIGSSKQGKIPT*
>lamu_GLEAN_10007413
MSPPLLGTGEEEGHSNVTVLTSSASADSLCQNGSELKERNYMGFSDCSSVGSSIVSAVSDESKNNLNLKATELRL
GLPGSQSPERDPEPRLLSSVQFDEKPLFPLHPSSDGHFSSTQKTIVSGNKRGFSDAMAGFSEVKTTAVTGRHTN
NFTEGLNLENIDTLMRGKFLSSSEVNVMLSPRPSPNLGLKPGSMLESLGAQSNKAKELVAQKVAQERPHATNEP
KQNHNSSTNNNSGPPATKAQVVGWPPIRSFRKNSLATTSKNTDEVDGKAGPGALFVKVSMDGAPYLRKVDLK
NYNKYQELSSALEKMFSCFTIGQYGSHGALGREMISERKLEDLLHGSEYVLTYEDKDGDWMLVGDVPWEMFID
TCKRLRIMKSSDAIGLVINFNGHFHTLTNFCPKGHGEMQEQELDRSEHVPHCPSSSLCSQALERMKKL*
>lamu_GLEAN_10007551
MGKSLAFENDDLNLKATELKLGLPGSDEPEKQSAAPTVRSNKRAFSPEISEKESKSSTNSSASDAAVDAEAATAA
KAQVVGWPPVRSNRKNNLLQKKNEADINGAGMYVKVSVDGAPYLRKIDLKVYKSYQEFLKALEKMFKLRIGEF
SERDGYNGSDYAPTYEDKDGDWMLVGDVPWDMFISTCKRLRIMRGSEARGLTSL*
>lamu_GLEAN_10007552
MDVGMKMAKMGGEVLEKEKMGYEETELRLGLPGNGGGSGSEGEGARKRGFSETVDLKLNLSSKEGLAADPT
EKPTKPLLNDSTLLPSATDSAKPPTKAQIVGWPPVRSFRKNILSVPKTSSEEGEKAGGNAAFVKVSMDGAPYLRK
VDLRMYKTYQELSDALCKMFSSFTIGNCGPQGMKDFMNESKLMDLLNCSDYVPTYEDKDSDWMLVGDVPW
EMFVESCRRLRIMKGTEAIGLAPRAMEKCKKRS*
>lamu_GLEAN_10009452
MGRASHSCSSSIDSSHVKHPFSATSSSSSSSWSNCPGAPNSGRMVTSPPAELDSRADLSTDLRLGLCISATDSAS
TQREQRSDWPPIKSLLRSTLNGKAAGNRRHASLFVKVYMEGIPIGRKLDLLAQDGYEALISTLRHMFRTTILYPD
GDRLHSDKHHVLTYEDKEGDWMMVGDVPWEMFVTTVKRLKITRADRC*
>lamu_GLEAN_10010984
MESSGKTNGKNGLNFKATELRLGLPGSESPDRDDADKNGDLVVLSLKSYVVSRAKRGFSDAIDGGGGSTKWVF
SSGSEAASVDLGAKGCSGGGGVLFSPRCTGKGVGGSDLGLGGPVVKDSVEPQSPKPVVQEKKAPAAAASSHG
VAPAAKAQVVGWPPIRSFRKNTMASHAPKNDDDAEAKLGAGCLYVKVSMDGAPYLRKVDLKTYGSYMELSSA
LEKMFSCFTIGQCGSHGVPSRDGFSESRLMDLLHGSEYVLTYEDKDGDWMLVGDVPWEMFTDSCRRMRIMK
SSEAIGLAPRAMEKCKNRN*
>lamu_GLEAN_10011061
MRGSPESDVTSMDMNFEDTELSLGLPGEGRLPSVTGRRNCLAKRGFLETVLELELSRSTLCAATSKPPSAKAQVI
GWPPVKSQRKKAVDRCQYVKVAVDGAPYLRKVDLQVYRSYQELLTALEQMFTIHGDYSDGEMTIVDRINGME
HVATYEDKDGDWMLVGDVPWKMFKESCKRIRVMKRSEAMGLAPRTPPKVRDPNEQETP*
>lamu_GLEAN_10011168
MELQLGLALPSNQINGFDLNSLVCEPKEGEGSNHFGCGVLQLGSSLFSGNGKKRGFEEAVEQRRTVPPTLPLLL
WDEQPNEKDDPRNLRSHSSLAVNKNDGESLVGWPPIKTHRKKVCHQNQRNRTWNNGAMENNGGGFRGR
ASNSMYVKVKMEGVGIARKIDLSLFHSLETLKETLISMFSTYHEDSTSYRLTYQDREGDWLLALDVPWRTFIRSV

QRLKLQRMSA*
>lamu_GLEAN_10011219
MGSERENYGMINFEETELRLGLPGGGVNDGEITPRSSGKRGFSETVDLKLNLSTKESKTEDSDKMKQKSVAPHP
SDLAKPPTKAQVVGWPPVRSFRKNVMTVQKTPADEAEKASGSGTSTAAFIKVSMDGAPYLRKVDLKLYKSYQE
LSDALGKMFSSFTIGNCGSQGMKDFMNESKLIDLLNGSDYVPTYEDKDGDWMLVGDVPWEMFVDSCKRLRI
MKGSEAIGLAPRAVEKCKNRS*
>lamu_GLEAN_10011220
MVIEGDVLNFEATELRLGLPGTSSEWEKQTGSPRVRSNKRASSEMNNGGSQVSDAKKSDEEAPPPTKAQVVG
WPPVRSYRKNCFQAKKAEEAATGIYVKVSVDGAPYLRKIDLKVYENYSDLLKALEEMFKFKVGKYSEREGYNGSE
YAPTYEDKDGDWMLVGDVPWDMFVNSCKRLRIMKGSEAKGLGSIAM*
>lamu_GLEAN_10013390
MSPPLLGVEEERQGNISLVSSSPSVDCISQNGSGLKERNYLGLSDCSSVDSSAVSSLPKDNKNNLNLKATELRLGL
PGSQSPEREPGLCLLSSGKLDEKPLFPLLPSKDGICSSSQKSVVSGSKRGFSDTMDGYSEVKGSVYTEKSWMFHA
AVSDSESPQSVGQGKYSGSSGINVMLSSRSSGVQPVIKKDIPQNMLQERSHATSGAKVTQNGASNNSSAPATK
AQVVGWPPIRSFRKNTLATTSKNNDEVDGKPGPGTLFIKVSMDGAPYLRKVDLRTYSTYQELSSALEKMFSCFT
LGQCGSHGTPGREMLSESKLKDLLHGSEYVLTYEDKDGDWMLVGDVPWEMFIDSCKRLKIMKSSDAIGLVDW
IIIILSNVSSLLSIHSSQSNGEIQEQKLAPTEKFESVKLLQSGKLCLSLYLISHHFSSWARPNFSFHTSLRMATVQVVN
FDTLVIGIALRETMAGNCMSVREVLIGMHVQAGLNFACSVQGSSHLFHSGFMVTPV*
>lamu_GLEAN_10013926
MGKAASSSSSSIESSNHFARSTGSSLNRDLSTDLRLGLSISTSDQPGGPLIPSEQPSDWAPIKPLLRQALTEEEYES
NMSPTFFVKVYMEGIPIGRKLDLLAHDGYHDMIRTLDHMFSTAILWAEEDGLHSEKTHVLTYEDKEGDWMMV
GDVPWE*
>lamu_GLEAN_10014915
MNSFDSQRQEPIRGRWQERRVLPNISNRFGAGDGGVSVITPPPCSPSMANTNVRFQTFPGFDDDDLVSAVVP
AVTVVLEGRSICQRISLHKHASYQSLAKALRQMFVDCGDAELSSETDLDLSNAVPGHLVAYEDMENDLLLAGDL
NWKDFVRVAKRIRILPVKRNPRKGRGGD*
>lamu_GLEAN_10016966
MELQLGLALPTTRSSPRGFDLNSLGFEQEEMVSSDTWNGGASLDSENYKIKRGYEAAFGDMRHDRSSRMVGL
LLWNGQPNEEDDRKGQRKSSDSSSINKNDEEESQVVVGWPPIKSWRKKLLPHHHQGVCRVGDNRMAMATR
ENNGSNSMYVKVKMEGVAITRKIDLKLYHSYQTLNNSLISMFPKYQKGYQDAANYVLTYQDKEGDWLLAGDVP
WQTFVESVQRLEILKNGIRSG*
>lamu_GLEAN_10017136
MEYSQSLDFIPEDQDWPVGDEVSEDKKLELRLGPPGQFHGYNNNNTHGAKRVFQQTVEANIADKTWLTSST
NNQCQRLNSIPLSCGSMPPSATKRETQKEILQSRPSFLQNQLISQKLGGMREHCSQLSRSRVACSSPQNTIETKS
SNIRIANPSGSSLICLSDCNLKTSRTSPAVAVGWPPVRSFRKNLASSSPSKMASESANKSLNEERAGKYDSSKDQL
FVKINMEGVPIGRKVNLSACSSYGELSFAIDKLFSGLLAAQRDCSADGNKLKEENATRSSLGRNGEYTLVYEDNE
GDRMLVGDVPWHMFISTAKRLRVLKSSELSTTGIGSSDQEKTPLDSAVEAGGR*
>lamu_GLEAN_10018461
MEGGLCLLGGVGSGGGGGGSSGGSSNDSTVSKLDGVEATEASSYPAESELELGLGLSLGGGGAGKSKASSAWGE
CGRILTAKDFPSVVCQSSRGASIGTKRAADSVSHEGGSPPGISQVVGWPPIGAHRMNSLVNQAKAPRAEEDNG
VGEKDKPKDASKMKIYSSDKINVAVNEKGHLGFVKVNMDGIPIGRKVDLNAHASYETLAHALEDMFFRSASTIR
PIGPCRDKEHSKLSRLLDGSSEFVLTYEDKDGDWMLVGDVPWGMFLSSVRRLRIMRTSEANGLAPRFQERNER
QRSKLV*
>lamu_GLEAN_10018587

MEVSRKMANMLGSDRELNLKETELCLGLPGGGASAAATEVEAPKATGKRGFSETVDLKLNLQSKEGAMDLNE
NMKAVSKEKNLLPAACAKDPAKPPAKAQVVGWPPVRNYRKNIMAQKSTGEEGDKSSGSGSGAAFVKVCMD
GAPYLRKVDLKTYKSYQELSDALAKMFSSFTMGNYGAQGMIDFMNESKLMDLLNSSEYVPTYEDKDGDWML
VGDVPWEMFVDSCKRLRIMKGSEAIGLAPRAMEKCKNRA*
>lamu_GLEAN_10018588
MESGVAYENDLNLKATELRLGPPGTDDNEEQGVSCVRGNKRSLLETSTEACGAKGKSDANHETAPPAKAQIVG
WPPIRSYRKNSFQPKTSEAEGPGIYVKVSMDGAPYLRKIDLKVYNGYEELLKALENMFKFTIGEYSEREGYKGSE
YAPTYEDKDGDWMLVGDVPWEMFMSSCKRLRIMKGSEARGLASGSPAGMFRRWRKAKKTKVVALQFLSSSL
TAKDIIKILSGLSICDVNVFLRNP*
>lamu_GLEAN_10019362
MQGGGGSTSGSMSTVSREENMVLSSEDSSCPEETELELGLGLSLGGGSFRGVQGGQYARILIAKDFPPVRSSSP
SSSPSSSSGSSSSSLSRANVTAGTKRTADSVAAANAGGNQVVGWPPIRAYRMNSMVNQAKSLATEELNSTIEK
NRSKSSIVEKSNTVGYKSNGNAKARTSLFVKVNMDGIPIGRKIDLNAHGCYVTLAKTLEVMFLKTSPPVDAIRSS
TQEHNTITVAAGPSKLLDGSSEFVLTYEDKEGDWMLVGDVPWVMFLSSVKRLRIMRKSEATGLGRKI*
>PoptrIAA3.1
MEFERDLNLEATELRLGLPGTATEQLEKQTPNSNVTKSNKRSLPDMNEDSAGRRESSSVSSNDKKSHEQETAPP
TKTQVVGWPPIRSYRKNCLQARKLEAEAAGLYVKVSMDGAPYLRKIDLKVYKGYPELLEVVEEMFKFKVGEYSE
REGYNGSEYVPTYEDKDGDWMLVGDVPWEMFINSCKRLRIMKESEARGLGCAV
>PoptrIAA3.2
MEFERDLNLDATELRLGLPGTATKQSEKQTPNSNLAKSNKRSLPDMNEEPAGSSRENSSTVSSNDKKSHDQETA
PPIKAQVVGWPPIRSYRKNCLQAKKLEAEAAGLYVKVSMDGAPYLRKIDLKVYKGYPELLKALEEMFKSKVGEYS
EREGYNGSEHVPTYEDKDGDWMLVGDVPWDMFINSCKRLRIMKESEARGLGCAV
>PoptrIAA3.3
MERSMAYERHLNLKATELRLGLPGSDEPEKPSTTPSVRSNKRASPEISEESRSKGSSSLSSNVENSEGDDAPPAKA
QVVGWPPIRSYRKNCLQPKKNDRVDGAGMYVKVSVDGAPYLRKIDLKVYRSYPELLKALEDMFKLTIGEYSEKE
GYNGSDFAPTYEDKDGDWMLVGDVPWDMFISTCKRLRIMKGSEARGLGC
>PoptrIAA7.1
MATATVLGTEMADLNYKETELCLGLPGAVGVKNEVETPNKATGKRGFAETVDLKLNLQAKEGVMDLNENIKNI
ASKDKNHLPADTIKDPAKPPAKAQVVGWPPVRSYRKNVLAQKNASEEGFRAQVVGWPPLRSYRKNVLTQKNA
SEEGDKASTGGSSAAFVKVCMDGAPYLRKVDLKMYKSYQELSDALAKMFSSFTMGNYGAQGMIDFMNESKL
MDLLNSSEYVPSYEDKDGDWMLVGDVPWEMFVDSCKRLRIMKGSEAIGLGMHLVISSYITIYNNLLLKFLNIIN
NSLLL
>PoptrIAA7.2
MTTSVLGTERTDLNYKETELCLGLPGAVGAKNEVETPNKATGKRGFAETVDLKLNLQAKEGVMDLNENIKNITS
KDKNHLPAVTIKDPAKPPAKAQVVGWPPVRSYRKNVMAQKNASEEGEKASTGGSSAAFVKVCMDGAPYLRK
VDLKMYRSYQELSDALAKMFSSFTMGNYGAQGMIDFMNESKLMDLLNSSEYVPSYEDKDGDWMLVGDVP
WEMFVNSCKRLRIMKGSEAIGLVSAPRAMEKCKSRT
>PoptrIAA9
MSPPLLGVVEEEGHSNVTLLASPASAESACLNGLELKERNYMGLSDCSSVDSSAVSAASDERKTSLNLKATELRL
GLPGSQSPERNHELSLLSSALLDEKPFFPLHPSNDGHYSSTQKNVVSGNKRVFSDAMDEFSESKFLSNSEVNAM
LSPRPSPNMGLKPGMLENLGVQQAKVKEIVAPKAGQERPHAANETRPLRNSSANNSSAPAPKAQVVGWPPIK
SFRKNSLATTSKNTEEVDGKAGPGALFIKVSMDGAPYLRKVDLRNYSAYQELSSALEKMFSCFTIGQYGSHGAP
GREMLSESKLKDLLHGSEYVLTYEDKDGDWMLVGDVPWEMFIETCKRLRIMKSSDAIGLGAFVMYLKPILLLF
>PoptrIAA11

MQGGFLGGGSGGSVCMSTVSMEDNVLMSSEDSSSPDEGELELGLGLSLGGASGFKDFGQRSSQQYARILTAK
DLPSKVSSSSCCSSTTSSSSSTLSRANATAGTKRAADSVSASNGAASSQVVGWPPIRSHRMHIMVNQAKSQATE
EFNSMNKRKNAVEEKVGNKNINIGNTKTRTSLFVKVNMDGTLIGRKVDLNAHGCYETLAQALENMFLRTTTTL
NMARLSTPEHKIMIDAKRHSQLLGGSSEFVLTYEDKDGDWMLVGDVPWGMFISSVKRLRIMRMSEATGLGK
>PoptrIAA12.2
MMAGGLGSLGGGGGSSGASTNDSTMSKVEVVEAEASSYPVEAELELGLSLGSGGRGGGGGGKGKANARGER
GRILTAKDFPSVVSQPQRPNNNTSISSACVVGAVSGTKRAADSVSHEGGSPTAGSQVVGWPPIRAYRMNSLVN
QAKAARAEEDKGIGEKDISKDNLKKKICNGNKTSAPSNEKGHLGFVKVNMDGIPIGRKVDLNAHACYETLAQA
LEEMFFRSATTINSIGGEKRQVTKPSKLLDGLSEFLLTYEDKEGDWMLVGDVPWGMFLNSVKRLRIMRTSEAN
GLAPRFQDRNEKQRIKPV
>PoptrIAA15
MSPENGSNLLESDAANVSFKETELTLGLPGESRGLALIEKTSGKRGFLETVDLNLGRSSNVDSDHNKYSGESETD
VPNTAKPPAAKAQVVGWPPVRAYRKNAMKSCKYVKVAVDGAPYLRKVDLEMYNSYQQLLNALQDMFSCFSF
TIRNYLNERTIMEQEVNNGVEYVPTYEDKDGDWMMLGDVPWKMFVESCKRLRLMKSSEATGFAPRTPSKCS
SSS
>PoptrIAA16.4
MEVEKGTKMGFEETELRLGLPGNGGGAEGEMVRKRGFSETVDLKLKLSSKESGADPNHEKTSSLQREKNLLAT
DPAKPPAKAQVVGWPPVRSFRKNMLAVQKSSTDQECEKVPGGNATFVKVSMDGAPYLRKVDLKMYKTYQEL
SDALGKMFSSFTIGNCGSHGLKDFLNESKLIDLLNGTDYVPTYEDKDGDWMLVGDVPWDMFVESCKRLRIMK
GTEATGLAPRAMEKCKNRSYK
>PoptrIAA19.3
MAQPLGLEITELRLGLPGSDDGHKNDKKRVFSEVSGEANSTTDDRKVQTKSQVVGWPPVCSYRKNISFNERDR
HHETSKIYVKVSMDGAPFLRKIDLGMHKEYSDLVVALERLFGCYGIGKALKDEYVPIYEDKDGDWMLVGDVPW
EMFFESCKRLRIMKSSEAKGFGLQPRGALKGISKDERH
>PoptrIAA20.1
MGRGAASSSSSSFESSRYPSVSGESSFPHVKRDLSTDLRLGLGISTSRQDNPSTPSEQLLDWPPIKPSPGKAVTSE
ENECCSSTLFVKVYMEGIQIGRKLNLLAHDGYHDLIQTLDEMFNTSILWPEMDVEHSGKCHVLTYEDKEGDWLI
VGDVPWEVFLPSVRRLKITRADSL
>PoptrIAA20.2
MGRGATSSSSSSFESSNYPSVSSKSSLSQLKKDLSTDLRLGLSISTSQQENPSTPSDQQLSDWPPIKPFLRKALASE
ENECSSATFFVKVYMEGIPIGRKLNLLAHDGYHDLIQTLDQMFNTSILWPEMDIEHSGQCHVLTYEDKEGDWLI
VGDVPWEMFLPSVRRLKITRADSL
>PoptrIAA27.1
MSMPLEHDYIGISSEVSSMENTSGTDTINISTTASKGLNLKATELRLGLPGSDSPERGNENQQLGFSLNNNNSKD
KSFVSGARRGFSVAIHGGSANWVFSGNAGSDPNFSLRGANSGKEGFPHSSKPVVQENKSQVDGANTNGHGA
APASKAQVVGWPPIRSFRKNTMASHLSKNDDGAEVKSGSGCLYVKVSMDGAPYLRKVDLKTFGSYMELSSALE
KMFSCFTIGQCGSHVVPGQDGLSESRLMDLLHGSEYVLTYEDKDNDWMLVGDVPWKMFTDSCRRLRIMKGS
EAIGLAPRAMEKCKSRN
>PoptrIAA27.2
MSSIPKEHDYIGLSETPSMEKISDKLSSSSSTLSTEENINSNSNSNSNSTNTSLNLKETELRLGLPGYQSPERKLTLP
AAGVSLFGKDIDTNNTNGYPLRPLKNLVSGTKRGFSDAIVGSSGKWVFSGSNGSEVDLGKGAILFSPRGDNGNS
QKSCVAGPAKKDDVAQSPKPVQEKISQVAAANENSSAPAAKAQVVGWPPIRSFRKNTMASSLVKNNEDVEGK
SGYGCLYVKVSMDGAPYLRKVDLKTYSNYLELSSALEKMFSCFTIGQCGSHGLRGQDGLTESRLKDILHGSEYVLT
YEDKDGDWMLVGDVPWDMFTNSCRRLRIMKGSEAIGLGRYSCKIFSSNCLTCLNSEHMTLIIHYGLFLRIQEPD

WMNLLSFCLYLHI
>PoptrIAA29.1
MELQLGLSLPTYNFIENFDLNNGGFEPKDQMLGSKPWISCEDGNYLDNKRSFEVAFEKNIKDASQELPLLLWSG
QPNDEDDWNGEKKISRSINKDDEEENQVVGWPPIKSWRKKVLHHQHQAGHVVNSTRMATAGNYEYGTGSN
SKYVKVKMEGVAITRKIDLRLYNSYQTLTKSLISMFAKCKNLEKDAARYSLTYQDKDGDWLIAGDVPWQTFMES
VQRLKIVRNAG
>PoptrIAA29.2
MELQLGLGLPSEKTMKGLDLNSYVSEPKGLLGSGQLQLGQYSWFSTNANDKKRSFIDAFEESSGNEDGPQTLPL
LVWNNQPNDEDDFPKDLDNHSSNSCASNKSDGESDWIVGWPPIKFKKKKLSRQSSRALEINRAVDNGYEDCQ
ARTSKYMYIKVKMEGVGIARKIDVSLHHSFPTLKQTLLDMFGICQENSSNYRLTYQDREGDWLLAEDVPWRNF
LGTVQLLKLMRRSS
>EgrIAA26B
MEGVSGNGVANPQLLDLMPNKREWHVMIDGAKARGSSEEKKLELRLGPPGTDDWCNLKQD
MNNKRRDEALLPFGHFPSMASTQIATNNGGHQARKFGSSEALASPWPGTSYKENCQKLQD
QEEMKPPRPYYQFPSTISQSKNSPFLPKETSQPNCNNKGLNLQDSGMKKAFSQASASASA
ANTAVPSVSQKRTAPGPVVGWPPIRSFRKNITSGSSTKPSPEPQNETPSKVGREDKPVET
CEKKGMFVKINMEGVPIGRKVDLKAYDSYEKLSSAVDELFRVLLAAQRDSSAGGINKKQE
EEKPITGVLDGRGEYTLVYEDNEGDRMLVGDVPWHMFVSTVKRLRVLKSSEVSALCLGTK
HDKNSLEMKD*
>EgrIAA15A
MELEKVKESPEVDANPGSSLEEITELRLGLPGENRGKSGTKRGFSKTVDLDASAVGRDDE
ANNSPVGGARVKDEVSGAAKPPAAKTQAVGWPPVKAFRKSVMKSCKYVKVAVDGAPYLRK
VDLEAYDSYQQLLAALEKLFSCFSICNYASERKIVDPANGAEFLPTYEDKDGDWMLVGDV
PWKMFVESCKRLRFMKSSEATGLGPTTQSACTSSS*
>EgrIAA28
MKMDLPKLKRMCYDLIAIEVCFLSVKKQVKELSPGIPPGPVVGWPPLGSFRKNIANNFLC
RQTSELSSNNFGRQDNCERQSEDPNQHMFVKIYMDGFPIGRKLNLKAYDSYEKLSVAINE
LFRGLLAAHSFTGDDTSAKREDTNAENGECTLVYEDNEGDRILVGDVPWNMFVSTAKRLR
ILKSSGHSASLPLVSSDRLSVVSSDQENKPLDSAAKTGK*
>EgrIAA29
MDLQLGLALPIHDPAKASEPKDHEGSYKKWVDNKRCHGEAFGEYSSGKLQLLAWSSGPNE
DDDLSAKRSKFSCSMNKFRYSEGADDDDQVLGWPPIKSWRKEFVHGQRPPHQDHYENVHH
AQKENGEPDHNMFVKVKMEGVAIVRKINLRTYRSYNSLKGALIAMFSRYNRDDFKDHASY
TLTYQDKEGDWLLAGDLPWLNFVESVHRLQIQRSRD*
>EgrIAA15B
MTMLTSLIFRGRAVIASLMMSSGGGVAQPVRRLATVAEDRLCKNGGNGKEKGLMRMKPKK
ENSKEGGKKKLSHLKNSFVILLPNLLMKKSEETVDDLHAQQKMPSSTETADGSSDSNASG
MDTELTLGLSRVGSCKAGITGAKRAYARTIGLGLGSGTGKQSGDTEVDAAENSAASQVSS
AGKVPASKAQVVGWPPVRSFRQKTLESCKYVKVAVDGAPYLRKVDLRTYNSYDELLKALE
GMFNCFTVNGNFLEGSKVMDPATRMEYVPTYEDGDGDWMLAGDVPWKMFAESCKRIRLMK
SSDAAGIAPRTPTCRG*
>EgrIAA33B
MLDGLMELQLGLALPCSDGYVRAQQQQHQQQQRRRHGLDLDLNVNGFGGGHGQGCDEDMV
EAVGSNRFDPGPSLETGCYYDRSEEECGYEYEENGNCERSVPRTLPLLFGGAPDDEVDGD

DDGEGHHRFSGNKESTYLKVTMEGVGIGRKIDLSLHRSLHSLMYTLINMFGLCYEESENL
KLAYQDRDGDWVLAENVTWRSFVRSAQRLKLLRMSG*
>EgrIAA33A
MDGFSSQRQDSMKRRFQDRSLPISSMDNYFAKVMAAAATPPSSSPAFLKNPNSNSNSNSK
IPSSIGFDDDLVAAVVPPVTVAIEGRSICHRISLHKHESYQSLAKALRQMFVDDADVPDK
DLNLSNAVPGHLIAYEDIENDLLLAGDLNWKDFVRVAKRIRILPVKTNSRKGRGEA*
>EgrIAA4
MAAQGEDLNLEATELRLGLPGTVESEKQQAPLSGRSMKRNLIDVNNEYGSNEEESNGSSA
QKCDKQDVHRPSKAQVVGWPPVRSYRKNCFQKKAEGESTGVFIKVSMDGAPYLRKIDLKP
YKGYSDLLKDLQDMFKFKVGDYCEREGYNGSEFVPTYEDKDGDWMLVGDVPWNMFITSCK
RLRIMKGSSEV*
>EgrIAA3A
MGSGGGGFEKDLNMEATELRLGLPGTASSTAVTDQEPEARQARNNKRSLVDRREEEASED
ASRALPKDHDSAPSSKAQIVGWPPIRSYRKNTLQTKKTEAETSSSGIYVKVSMDGAPYLR
KIDLNVYKGYSDLLKALQNMFKFTIGEYSEREGYKGSEFAPTYEDKDGDWMLVGDVPWEM
FMTSCKRLRIMRGSEARGLGCAV*
>EgrIAA16
MSAETAERFTIDFEETELRLGLGRPAGVSSNGEGATRSGGKRVFLETVDLKLNFSSKEDG
GSVEKIRASAPAEKKMNSSDGKEKSVAAAAAAAAPPPPSSSSEVAKPPAKAQVVGWPPVR
SFRKNIMAVQKSGSDEAEKGGSSGNGAATSGAAFVKVSMDGAPYLRKVDLKLYKSYQDLS
DALAKMFSSFTIGNCGSGGMKDFMNESKLIDLLNGSDYVPTYEDKDGDWMLVGDVPWDMF
VDSCKRLRIMKGSEAIGLAPRAVEKCKNRS*
>EgrIAA13
MEAPPARGREAAAPKRDSAGEEAELELGLGLSVGGGGGGGERAGAKRGRILTARDFPSSV
GTKRTADESVSQEGGGGSPTSASQVVGWPPIRAYRMNSLVNLAKAPRAEDNMSPNEKSKS
KDGSEDNTRTGGMTDVDGREQKHIGFVKVNMDGIPIGRKVDLNAHACYETLAQALEDMFF
RPAKTIDLTGAEENRQVKKSSKLLNGCSEFVLTYEDKEGDWMLIGDVPWGMFLTAVKRLR
IMRTSEVNGIAPRFQQKSERQMRKPI*
>EgrIAA3B
MEFREMERGVGDGVFGKDLLNLEETELRLGLPGTEESGQKKSRTGKRLFSESSDVSGSSK
GSCVAPHHDEDHESAPAPKAQIVGWPPVRSYRKSALQPKKAEAEGPGIYVKVSVDGAPYL
RKIDLKVYGGYPELLKALENMFKLTIGDYSEREGYKGSEYAPTYEDKDGDWMLVGDVPWE
MFILSCKKLRIMKESEARGLGYGV*
>EgrIAA31
RTRRGDLNTDLRLGLSIVSPDAQKDNWIGPSVSKQREQRQDWPPIKSLLRTTLAVAGKAD
TNVHNVHNNDNSDRRRTSLFVKVYMEGVPIGRKLDLLTLHNYDDLISTLAHMFTTSITCP
NGDRADHAKSLHVLTYEDREGDWMMVGDVPWEMFMTTVKRLKITRADRC*
>EgrIAA9A
MSPPLLGVEEGGGNTSTVATSPSIDGASHDCLGLKERNYLGLSDCSSVDSSAVSSLSDEN
KSNLNLKATELTLGLPGSQSPEREPKLCLLSSGKLDEKPLFPLLPLKDGICSSSLQKNIA
SGNKRGFSDTIDEFSELKSSKYPDGNWMFHATGPAPETAQCGGQGKFPGNAGMKAMLPSR
TSGAQATVPKEALPKPAPECPRALNGAGVSQTRASNNAPAAKAQVVGWPPIRSYRKNTLA
TTSKDNDEVDGKPGPGALYVKVSMDGAPYLRKVDLRNYSTYQELSSSLEKMFSGFTIGQC
GSNGTPGREMLNESKLRDFLHGSEYVLTYEDKDGDWMLVGDVPWEMFIESCKRLKIMKGA

DAIGLVNK*
>EgrIAA19
MGKRGAVGLEIMTELRLGLPGAAKPSSNKRVFSEISTTEEKGSTEEPKAPAKNQVVGWPP
VCSYRQRNSFNKEKVDRAEAGAGSKAYVKVSMDGALYLRKIDLSMQKGYSDLAFSLEKLF
GCHGIGEALNGGEGAEYKLIYEDKDGDWMLVGDVPWEMFMESCRRLRIMKRSEAKDFAIL
QTRDFLKGTLKDDN*
>EgrIAA26A
MERVSRNGEASQLLDFVAKEGEWLMKRLQEQRRGPTPPEDKALELKLGPPGDEDRSKKDF
TMKYKLEKDESLLSLGYFNGGNQAQAQNLAPYLQLSPSIFRTQLPVLAKDKSSETVDLQN
NNDAAEKKRAFLPASAAATTAVPNSSQKRTAPAPVVGWPPLRMFRKNLTSSDGHGKPAPK
THQNMLSSKIVSEKPAESSGKGLFVKMNMDGVPIGRKVDLNAYDSYEKLSAAVDELFRGL
LAAQRDSSGCDIKSTQEEEKPITGLLDGRGEYTLVYEDYEGDRMLVGDVPWHMFISTVKR
LRVLKSSEISVLSAVQNVLAAGGQKQDKMSSDSAMQ*
>EgrIAA27
MSTPLVHDYIGLKEASLMSRSSEKALPSSSAGEDEKKSALNLRETELRLGLPGSLSPERK
QALGVPLFGKDLESKSGVLGFALSPSKNSVSGAKRVFCDAIDGCPSKWVFSASNGKSEVD
LAKGGPVLSSPRSGKESDNGVNALQSCVPKPAMTEGLGGVPQSPKPEQEERKNQGAGGSE
HGSAPAAKAQVVGWPPIRSFRKNTTASTLAKNCDDAEGKCLYVKVSMDGAPYLRKVDLRT
YGSYSELSTALEKMFSCFTIVSGHCDSRGPLGQESLSESRLADLLNGSEYVLTYEDKDGD
WMLVGDVPWEMFTDSCRRLRIMKSSEAIGLGTDSLQHVQVFIYQAIHLL*
>EgrIAA9B
MSPPLLSVGEEECESNVTLLASPSSLGSVCQNAELKERNYMGMGDHPSADGSVIPNAFDG
CKITLNLRATELRLGLPGSQSPERDADFCTISSAKLDEKPFFPLHPSNDGHYSSQKIVVS
GNKRGFADAMGGDSEAKFMANPEVNVVLSPRPSPNLGLKNGSGLESFGSRPAKVKEIPTA
QVAPEKPYSTKETQPNNGSRSNNAGSPAPKAQVVGWPPIRSFRKNSLATTSKNAEEVDGK
LGSGALFIKVSMDGAPYLRKVDLKHYSAYQELSSALEKMFSCFTIGALGREMLSESKLKD
LLDGAEYVLTYEDKDGDWMLVGDVPWEMFLDTCKRLRIMKSCDAIGLAPRAVEKSKNKN*
>EgrIAA32
MDRNAPAGFVFNPSSSHSMYYQDKKGSRMIDLGLSLRTLQSDSCDDPSGDSMTTHGCGEL
FDWQQLNQLQYSNSKNPCSKILQDAYYDEEAEGVQSEERSIYVKVTMDGLVVGRKLCMLD
HSSYCSLALQLEDMFGGQCAYGLRLSEPESEYTLHYKDREENWSTVGDVPWMEFIGRVKR
MRITRKSEAFLPMILERHQPTNNQLNWGFESNDSTEM*
>EgrIAA17
MMEVGLKMGGKLMQSEERDQKKKHEDAEAEERETELRLGLPGGNNGGSGSDQAPDLQVVV
GARKRGYAETEVDLKLNLNLSSSSSSSGSPPPSSGSDPNDQTNSLPGRLDKRNLLPCPTS
DIPVKPPAKAQVVGWPPVRSFRKNMVAAQKSSTEDMSSGGAASFVKVSMDGAPYLRKVDL
KMYKSYQELSEALCKMLSSFTIGRCESQGVKDFMSESKLRDLLNGSDYVPTYEDKDGDWM
LVGDVPWEMFVESCKRLRIMKGTEAVGLAPRAMEKCKSRS*
>EgrIAA20
MGKGSSSSSASSSTASSMSRRLKKRLTTDLNLALGFSPASHQDGGYPFSPKEAIEEEGDE
CDNAATFYVKVYMEGIPIGRKLDLMAHGGYCDLIRTLDYMFNTNIMWSEEEGAQCGGCHV
LLYEDKEGDWLMVGDVPWEMFLSSVKRLKITRIE*
>EgrIAA1
MEGSKSNGYGECDQANLKATELRLGLPGTEEEPPLKVESPSFNSRKRALADDAESVLQDG

RKSTSGVPPPSKAPIVGWPPVRSYRKNIYSSPAEVAEGGAGAGYLKVSMDGAPYLRKVDL
KTYKSYQELMESLENMFNITIGDCSDKGSDYAPTYEDKDGDWMLVGDVPWNMFTSTCKRL
RIMRGGSRS*
>EgrIAA11
CLLLLLLLLLLLLLLAFKFTSLSKVLSLSLSKRVLLRISASLSLSLYLPSSAVEEMQGDS
AGEAASPAAELTGSKGDDDYVASSSEGSSTPDELALGLTLGVGDSRPFKSPRPGPVILTA
KDLPSFVPASPSPPPPPLPASASASSSSSCSSTLSRAEGGAGTKRGADSVAAPTASSQVV
GWPPLKTHRMNVFINTPKSTSTGEFDPVVEKNDSKLAVLEKINNARNDKSSNSMKVSCPK
TSPFVKVNMDGVAIGRKVDLNAHQCYESLAETLEDMFDHPTTKVNAPRSNRLMYHGLVRA
TSASKLLDGSSGYVLTYEDKDGDWMLIGDVPWRYVARWYFHFDCLYCVLPYASPAPIHFG
IWHFSYLSFEAQVEIFNHSC*
>OsIAA1
MSVETERSSTESSAASGLDFEDTALTLRLPGSLAAAAAPDPDRKRSSPSSSDAADAADNSSPLAAAADAP
PAPKARVVGWPPVRSFRKNALAAKFVKVAVDGAPYLRKVDLEAYSGYDQLLRALQDKFFSHFTIRKFADD
ERKLVDAVNGTEYVPTYEDKDGDWMLVGDVPWKMFVETCQRLRLMKSSEAVNLAPRAAQ
>OsIAA2
MAWRRGFGREEEDAAAAGESGLELCLGLPAYFSSSSSSKPSEGSTAAPAFALRSNGTNASKPSGAAAAAP
VVGWPPVRSFRRNLASSSSSSSKQAPPPPSSSPQNGDKASKDGGAEKGMFVKINMDGVPIGRKVDLAAYG
GYAQLSAAVDKLFRGLLAAQSAAADGEADAAAAGEMVGGGEYTLVYEDDEGDRMLVGDVPWQMFIATAKR
LRVLKSSDLPPPSLMRAAGSRKRAAADS
>OsIAA3
MSPPLELDYIGLSPPPPPPSSSSAAAARADDVDLKGTELRLGLPGSESPDRRPAAIAAAAATATTLELLP
AKGAKRVFPDEAALTPPTAAAGKGKAAREGEEVGAEEEDKKVAAPPQPAAKAQVVGWPPIRSYRKNTMAT
NQIKSNKEDVDAKQGQGFLYVKVSMDGAPYLRKVDLKTYKNYKDMSLGLEKMFIGFSTGKEGAENQKDGE
YVLTYEDKDGDWMLVGDVPWEMFTDSCRRLRIMKGSDAIGLAPRAGEKSKNRN
>OsIAA4
MEECKGGGMSPSSSMDSSTHPALSTTSSAATARRDLSTDLRLGLSLSTSSSSSLLQAAAAAAAADDSIPS
TPRNSQVHADWPPIKPFLRSALQKASAAGGGGARRRRTLFVKVYMEGVPIGRKLDLLLLDGYDSLLIKLC
HMFKTPITYADVMECHQQVPGQKAAHVLTYEDQDGDWMMVGDVPWELFLSSVKKLRIARMDKC
>OsIAA5
MSPPLEPHDYIGLSAAAASPTPSSSSCSSSPNPGGEARGPRLTLRLGLPGSESPEREVVAAGLTLGPLPP
TTTKAASKRAFPDSSPRHGASSGSVAAAAACQDKAAPAAAPPAAKAQVVGWPPVRNYRKNTLAASASKGK
GEDKGTAEGGPLYVKVSMDGAPYLRKVDLKMYSSYEDLSMALEKMFSCFITGQSGLRKSSNRDRLTNGSK
ADALQDQEYVLTYEDKDADWMLVGDLPWDLFTTICRKLKIMRGSDAAGIAPRSIEQSGQSR
>OsIAA6
MEEGSNKREGLPPQLLDLIPDEKEWKLREALGLGRSRNAGFDGEEDKKLDLKLGLPGFIEDDEAETLRDY
RLQQESPSLSLSFFPKHSKTTSSTTTTTGAKRGFIDTVEDKTEGYNDQKQQARAGCGKELAVEEMIAAVS
ERKKGCCPPPPPPHGAPATPARNRPQTQGRGAAAPVVGWPPIRSFRRNLASSSSSKHSPEPQNDNANAKV
TLTCKKNPLVKINMDGIPIGRKIDLAAYNSYDGLSSAVKQLFHGFLQAQKDQTNAQIAQQGADDKIFYQL
LDGSGEYTLVYEDSEGDRMLVGDVPWKVFVSTAKRLRVLRSSELSHTLIGATARV
>OsIAA7
MGEASESMKKISRGRLGGSWMGEPSDHHRHGDEQEEEEKTLELSLGLPGGGWRAACRDKGTTTKHSIAAA
AAADDDDGDKSSMLSLGYSTLVSHSQGKANKNKGSPEEEEAHPPPATGNNALASNNNGCFQTRSPSTPVV
GWPPVRTFRRNLATSSKASLELQNGKKAAKAEEIKRAPFIKINMDGVPIGRKIDLNAFDSYEKLSLAVDK

LFRGLLAAQRDPLTAGAKDCQQEDVAISGLLDGTGEYTLVYEDYEGDKVLVGDVPWGMFVSSVKRLRVLK
TSDLSSSLITSGRKRTAAEC
>OsIAA8
MECMASTEESLPASSSMDSCSGELPTTTTTAPAQSTASSGCRPPATAAKRRSLISTDLRLGLTLSSVVHI
DGNNPSTPRSSLTTATVTADRGGGGGGHGRRRSLFVKVYMEGVPIGRKLDLLPLDGYKGLVARLASMFRA
SITYHHCHRQFAVVGMKTNKVHHVLTYEDQEGDWMMAGDVPWELFLTSVKRLRIARADDKYCYSC
>OsIAA9
MELELGLAPPNSGHLVVDELSSSSSSGGGSGSAPVSASSAGKRGFREAFQETLLLFDDGSCCNTSDDDCR
RRKKTVVGWPPVSSARRACGGANYVKVKKEGDAIGRKVDLALHSSYDELAATLARMFPTNDHQGEKKMAN
DDHGDAAGPVVTYEDGDGDWMLVGDVPWDDFARSVKRLKILG
>OsIAA10
MRGGVAGPTAGEPPGTEAEAEEVEESSAGDDEELELGLSLGSKKQQQQQHAPCRILTARDLQPAAALSPD
SSVSSSSPAAAAAAGGKRAEGPTATTSPGTVASGHPHSSFGVVGWPPIRQFRMNSLFNQAKENTSETDTK
KTATNESDVQKDKEEGEKKGRVAGWVKVNMDGEVIGRKVDLNAHRSYKTLALALELMFTKPSIGLCASHN
TNSLKLLDNSAEYQLTYEDRDGDWMLVGDVPWEMFVSSVKRLRIMRTSDANGLGQRYQGIHRTIASTRGR
S
>OsIAA11
MAGLGFDETELRLGLPGAGELAARSSGKRGFAETIDLKLKLQPAAPAAVSGEEGAQEDKEDADAAAAAAD
EKMSMKRSASQSSVVTAEPDPDKPRAPKAQVVGWPPVRSFRKNVLAEKCKAAALVKVSMDGAPYLRKIDV
AMYKSYPELSMAFQNMFTSFTIGKCGSHQQLKESNKLRDDLEYVPTYEDKDGDWMLVGDVPWEMFVESCK
RLRIMKGSEAIGLAPRAVEKCKS
>OsIAA12
MEAAVGYAADSLIKATELRLGLPGTADDLPSTPRGKKRAAAAEDNNANAAAADDDEHDAVEAAPPVAKAQ
VVGWPPVRSYRKSCFQQQSAAASKSKAAVSSCNNKDEPITKNAAPAPAASSAAAANGGSLVKVSMDGAPY
LRKIDLRMYKGYRELREALEAMFVCFSGAADGANPSEFAITYQDKDGDLMLVGDVPFDMFTSTCKKLRIM
KRSEATGLGSPRQMKI
>OsIAA13
MAGADVDVGTELRLGLPGGGGGAAEAAAKAAKRGFEETIDLKLKLPTAGMEEAAAGKAEAPAAEKAKRPA
EAAAADAEKPPAPKAQAVGWPPVRSFRRNIMTVQSVKSKKEEEADKQQQQPAANASGSNSSAFVKVSMDG
APYLRKVDLKMYNSYKDLSLALQKMFGTFTATGNNMNEVNGSDAVTTYEDKDGDWMLVGDVPWQMFVES
C
KRLRIMKGSEAIGLAPRAKDKYKNKS
>OsIAA14
MAAESIDAELRLGLPGSGGGDGVAAKKRRSASSTVKSEASGTACCGGAGARDVEDGASPASKVQVVGWPP
VGSYRRSTFQSSSSSTAAAAKGKGGGETDQGRKNKGGGLYVKVSMDGAPYLRKVDLRMYGGYRELRDALD
ALFGCFSADASASAAHFAVAYEDKDGDLMLAGDVPWDMFISSCKKLRIMRGSEAR
>OsIAA15
MSVETERSSTESSAASGLDFEDTALTLRLPGSSSSSSSSSSSSSSSPSEPDRKRASATDDDPDNRLGST
ATESPPSPKARVVGWPPVRAFRKNALAALAAASSSKAKFVKVAVDGAPYLRKVDLEAYRGYDQLLAALQD
KFFSHFTIRKLGNEEMKLVDAVSGNEYVPTYEDKDGDWMLVGDVPWKMFVETCQRLRLMKSSEAVNLAPR
SA
>OsIAA16
MAWNGRFGEDGEEERSLELSLALPGYFSSSGLQGNTSTAADGAKGNDGFKASRPAAPVVGWPPVRSFRRN
LASSSSSSKPPRGGRDAAAAATGGKVARFVKVNMDGVPIGRKVDLAAHGGYGELSAAVDRLFRGLLAAQR

DPTMATAAAAAAAAGESCTGEEEAIAGLLDGGSGEYTLVYEDDEGDQMLVGDVPWNMFIAAARRLRVLRSS
DLNASTIRAGSRKRAAAE
>OsIAA17
MSPPLELDYIGLSPPVPAAADAAADNDLKGTELRLGLPGSHSPDRSPPAATLDLLPAAKGAKRGFSDEAR
PLPASAAAAAAAGKGKKAAAGEEDEDAEEEDKKVAAAPQAPAAKAQVVGWPPIRSYRKNTMATNQLKSSK
EDAEAKQGQGFLYVKVSMDGAPYLRKVDLKTYKNYKDLSTALEKMFIGFTTGKDGLSESRKDGEYVLTYE
DKDGDWMLVGDVPWEMFANSCRRLRIMKGSDAIGLAPRAVDKSKNRN
>OsIAA18
MEEEFKDKGLPPTLLHLIPDGREWKVKEADGEGSRNTNLDADEDKELELKLGLPGVQQEERAADSREKIQ
QQQRESSSEPSIGCFPTHSKPTTSIGTTGAKRGFFAIVGATLEGYNQSHRDTEECGKELTLGDENMAGER
KKGCCPSPPCSAAAHSSNPQGRGAIPPVVGWPPIRSFRRNLTNGSSFKQSPERQNDEADDKAKPICKKRP
LVKINMDGIPIGRKVDLQIYDSYQKLSSAVEELFRGFLEAQKDLSCAESGEQGAEDKIFSGLLDGTGVYT
LVYEDNDGDRMLAGDIPWKVFVSTVKRLRVMRRSELPHDMIGADPVK
>OsIAA19
MPPPLEARDYIGLAASPASSSSSCCASTPVAEVVGAHLALRLGLPGSESPARAEAEAVVVDAALTLGPAP
PPRGGAKRGFVDSLDRSEGRRAAATAGDDERGVREEEEEEKGLGEAAAGAPRAAKAQVVGWPPVRSYRKN
TLAASATKTKGEDQGKSEVGCCYVKVSMDGAPYLRKVDLKTYSSYEDLSLALEKMFSCFITGRSSSHKTS
KRDRLTDGSRADALKDQEYVLTYEDKDADWMLVGDLPWDLFTTSCRKLRIMRGSDAAGMAPRSLEQTGQN
K
>OsIAA20
MELELGLRLALPSPSPSPATATAAGSELDLLNSAPGSCRKRGFEEALGGFKTDDDNDDGNGRGGDGDSDG
EMGNKRRKLVGWPPVKCLHRRRDGGCGGGYVKVKMEGLAIGRKLDLSILGSYAELLDTLHLMFPSTNQED
GHDRRRRHPYAVTYEDGEGDWMQVGDVPWEAFAKSVKRLKILV
>OsIAA21
MAPPQERDYIGLSPAAAAALATELRLGLPGTAEEAESEGGGGGGTDAAPLTLELLPKGGAKRGFADAIVG
GPAGQRREAAGGKAAAAAAEAEEEEEKKKAQAPAAKAQVVGWPPIRSYRKNTMAMSQPALKGKDDGEAKQ
APASGCLYVKVSMDGAPYLRKVDLKMYKNYKELSLALEKMFSCFTVGHGESNGKSGRDGLSDCRLMDLKN
GTELVLTYEDKDEDWMLVGDVPWRMFTDSCRRLRIMKGSDAVGLAPRATDKSKNRN
>OsIAA22
MKLKAAAVVSCDFGKGKLYPQVMGAGWNESGENRAASSAQLVGWPPVRTFRKNLSTPKPADADDLMNKM
K
PCSDEGHGSRDAAQERRPSSTMFVKVNLEGYAVGRKIDLKAHRSYDSLSQALQSMFHGFLSDGIATRDNE
LQRMEEGSKKRYVLVYEDNEGDRMLVGDVPWDGREAGSGRRPAAGVDQVSERPDVSPAMATTPAATVAVT
QRRELTNLAATVAAHVLVFLASGQGHINCMMHFAMGDIVELLESLGTNGSLVKGD
>OsIAA23
MSTSSGADSSPPVSGLDYDDTALTLALPGSSSSSSSTADPERKRAAHADHADAKPPSPKARAVGWPPVRA
YRRNALREDSARAKLVKVAVDGAPYLRKVDLAAHAGYAPLLRALHGMFASCLAVRGGGGGDGEGTKLVDL
VTGAEYVPTYEDKDGDWMLVGDVPWKMFVESCKRIRLMKSSEAVNLSPRRSSR
>OsIAA24
MASSSSLRSTSCLASAAETDADNLCLRLGPPGSSITTTTTTGGADPAAKRSLGAKRSLESTDSMASGTGT
SAAGDEHDDDTAAPAKAQVVGWPPVRAYRRNTFHQAAAAAAATKKGGDEKQKQQQQGGGLYVKVSMDGA
P
YLRKVDLKMCKGYRELREALDLLFTKCFSATASDGCSDGQFAIAYEDKDGDLMLVGDVPWEMFISSCKKL
RIMKGSEAR

>OsIAA25
MKSSSVAPRLKQERQDDCKFQEGDVNSLELRLGISSDNDQISGGGAASPWLGVGVHPWSLAARQGKAALE
QAHQRPNECAVQRENRAASSAQLVGWPPVRAFRKNLSTPKPADADDLMNKVKLCSDEGHGSRCAAQERRS
SSTMFVKVNLEGYAVGRKIDLKAHRSYDSLSQALQSMFHGFLSDGIATRDNELQQMEEGSKKRYVLVYED
NEGDRMLVGDVPWELFIASVKRLYIAQDPRVHAKLR
>OsIAA26
MASYGDDGVELTELTLGPPGASARRARRGRKNGHPPPSSSMIQAAYFVKVSMDGTPYLRKVDVAAYGDYL
ELVEALNDMFYCSTIGLMDGYGEWEHAVVYEDGDGDWMLVGDVPWEMFVSSCKRMRVMRACEARGLSSN
A
>OsIAA27
MMNLISFETPPLGRRSQDGGSSSSSITAATTTTNKAKEAASHLDLSLGISLSPGGGGGDAGTKASSCCYG
GGGDGGGCMGSGMLTAGVLGVGHGGSSHDNTTASSGGGGSWTAAFMPSPTGFMHPWSLAARQQKAAAE
QE
RSGVARLPPATTTYMPRAAATVISLPAAVGWPPVHTSRRNLVATINNVLKPDTTAAVKPDRPTQATAMFA
ADETTAPPPRSAAAATEASRTLNMFAKVHMDGYKVGRKINLRAHRNYDSLRRVLTKMTHNFFCPADYSST
NKGEEDCAKSDEFIFLYEDFEGDRMLVGDVPWELFLASAKRLYIAKNPAPRNKGTYRPLVWICMLLPKAP
Y
>OsIAA28
MGRMKDRNASAGPEVKPAGLSPSRFVKVFMHGEPFERKINLAIHNNYDSLSFTLKRLGNNYSMSPFELEG
FVNNEEDGAIDNDFDLLYDDMNGVRYLLGEVPWEVFTITVKRIYIVPAEQQNESEYQEEEDNAAAAATA
DEDVDGNHWWRNHLWSVGPILQ
>OsIAA29
MKDRNASAEPVVKPGLSPSRFVKVFMHGEPFGRKINLALHNNYDSLSFTLKKLGNNYSMSPFELEGLVNK
EEDGAIDSDFDLLYDDMDGVRYFLGDVPWEVFTTTVKKIYIVPAEQQNENDYQEEEDNAAAAATADEDG
DGAAADDGVAAAADDVDDVAGYTSNDDPSFD
>OsIAA30
MAADLAFEATELRLGLPGGGGDGDAAAAAARSSSGKRGFAETIDLKLKLEPAAAAVDDDDDKEEAAADDR
EKKVDIVGADNDDASPPAAAAAGGMKRSPSQSSVVTAAADPEKPRAPKAQVVGWPPVRSYRKNILAVQAD
KGKDAADGGGDKSGAGAAAAAFVKVSMDGAPYLRKVDLKMYKSYLELSKALEKMFSSFTIGNCGSHGVNG
MNESKIADLLNGSEYVPTYEDKDGDWMLVGDVPWEMFVESCKRLRIMKGSEAIGLAPRAMEKCKNRS
>OsIAA31
MENLKATELRLGLPGTEEEAAPPPSTPRAGSKRALAGEPDQAKIKPAAAAKAQVVGWPPVRSYRKSCLQP
TTTTTKSKPPPAAAAAETQQKEDVAGAGGLFVKVSMDGAPYLRKIDLKVYKGYRELREALEAMFLCFSGG
AAADAAVNPSDFAVTYEDKDGDLMLVGDVPFEMFISTCKRLRIMKGSEARGLGATRG
