# Supplementary material for: Genome-Wide Identification and Expression Analysis of the Aux/IAA Gene Family of the Drumstick Tree (Moringa oleifera Lam.) Reveals Regulatory Effects on Shoot Regeneration
Source: Int J Mol Sci. 2022 Dec 11;23(24):15729. doi: 10.3390/ijms232415729 (PMC9779525; doi:10.3390/ijms232415729)
Supplement: Supplementary file 1 [file ijms-23-15729-s001.zip › Supplementary files caption.pdf]

Supplemental Figure S1. Relative expression level of *MoIAAs* under NAA treatment (0, 0.1, 0.5 and 1 mg/L) at 6, 12, and 24h. The expression value was calculated relative to 0h.

Supplemental Figure S2. The structure of *MoIAAs* recombinant vector.

Supplemental Table S1. The qRT-PCR primer of *MoIAAs*.

Supplemental Table S2. The CDS-primer of *MoIAA1*, *MoIAA7* and *MoIAA13*.

Supplemental Table S3. The sequences of Aux/IAA proteins.

Supplemental Table S4. The information of MoARF proteins.
